# Nutritional Strategies for the Prevention and Management of Cow’s Milk Allergy in the Pediatric Age

**DOI:** 10.3390/nu15153328

**Published:** 2023-07-26

**Authors:** Serena Coppola, Laura Carucci, Franca Oglio, Claudia Di Sarra, Gulsum Ozen, Roberto Berni Canani

**Affiliations:** 1 Department of Translational Medical Science, University of Naples Federico II, 80131 Naples, Italy; serena.coppola3@unina.it (S.C.); laura.carucci@outlook.it (L.C.); dr.ogliofranca@gmail.com (F.O.); disarraclaudia@libero.it (C.D.S.); ozen_gulsum@hotmail.com (G.O.); 2 Immunonutrition Lab at the CEINGE Advanced Biotechnologies Research Center, University of Naples Federico II, 80131 Naples, Italy; 3 European Laboratory for the Investigation of Food-Induced Diseases, University of Naples Federico II, 80131 Naples, Italy; 4 Task Force for Microbiome Studies, University of Naples Federico II, 80131 Naples, Italy

**Keywords:** food allergy, gut microbiome, immunonutrition, Mediterranean diet

## Abstract

Cow’s milk allergy (CMA) is one of the most common pediatric food allergies. The prevalence and severity of CMA have increased dramatically in the last decades, under the pressure of environmental factors in genetically predisposed individuals. Among the environmental influences, nutritional factors play a crucial role. Diet is the most modifiable factor, representing a potential target for the prevention and treatment of CMA. In this review, we report the most scientific-based nutritional strategies for preventing and managing pediatric CMA. In addition, we propose the most complete supplement of compounds able to prevent nutrient deficiencies in CMA pediatric patients and to positively influence the disease course.

## 1. Introduction

Cow’s milk allergy (CMA) is one of the most common pediatric food allergies (FAs), affecting up to 3% of the children population. CMA derives from a breakdown of immune tolerance against cow’s milk proteins (α-lactalbumin, β-lactoglobulin, serum albumin, caseins, bovine serum albumins and others) that generally occurs in the first years of life [1]. Commercial milk is subjected to a heat treatment process to make it sterile, which through the Maillard reaction can induce the formation of deleterious compounds, such as the advanced glycation end products Nε-(carboxyethyl) lysine, Nε-(carboxymethyl) lysine, pentosidine, pyrraline, methylglyoxal-lysine dimer, glyoxal-lysine dimer and argpyrimidine, that may play a role in the pathogenesis of allergies [2]. CMA may present different phenotypes based on the immune mechanisms: IgE mediated, non-IgE mediated or mixed [1]. Infants with IgE-CMA may present from gastrointestinal (i.e., vomiting and diarrhea), cutaneous (i.e., erythema, urticarial and angioedema), respiratory and/or systemic symptoms up to the most severe reaction anaphylaxis, occurring within 2 h after cow’s milk exposure. Non-IgE-CMA is characterized by subacute or chronic gastrointestinal symptoms and affected infants may present bloody stools, delayed vomiting, crying and chronic diarrhea with the risk of malnutrition and failure to thrive [1]. The diagnosis of CMA requires a positive oral food challenge to cow’s milk protein; that is the gold standard test to confirm CMA [3,4]. The type and the severity of CMA symptoms dramatically worsened in the last years, as well the epidemiology picture showed an increase in CMA prevalence and persistence [5,6]. Several hypotheses have been postulated to explain the spread of CMA in the last years and the most likely one seems related to an impaired gene-environment interaction [6,7]. Some dietary habits (i.e., the Western diet), infections, cesarean delivery, formula consumption in the first week of life and the massive use of drugs in the first stage of life have been proposed as the main environmental factors responsible for the occurrence of FAs, including CMA [8,9,10]. Indeed, operating on an unfavorable genetic background, these factors impair the gut microbiome (GM), with consequent alterations of the GM-immune system axis. The perturbation of this axis could lead to a breakdown of immune tolerance and to CMA occurrence [9,10]. On the other hand, the Mediterranean diet, vaginal delivery and breastfeeding could positively modulate the axis and may represent an innovative approach to prevent and treat GM-immune system-derived diseases, such as CMA [9,10,11]. Among the environmental factors, diet represents one of the main modifiable ones. Since dietary habits have a role in eliciting potentially negative or positive effects in CMA occurrence, nutritional modulation could be considered an effective target for the prevention and management of CMA. The potential to influence the immune system functionality of selected dietary habits has been described with the term “immunonutrition” that, in the FA field, is based on a proactive approach focused on the prevention and the acquisition of immune tolerance in allergy treatment [12]. In this review we analyze the nutritional strategies from different points of view, covering different aspects of an effective approach to prevent or manage CMA.

## 2. Cow’s Milk Allergy Preventive Nutritional Strategies

The alarming increasing rate of CMA prevalence advocates the necessity for effective preventive nutritional strategies against the disease burden.

The CMA’s primary prevention should start from the prenatal period, focusing on a maternal healthy lifestyle and food diversity during pregnancy [13]. Maternal diet during pregnancy has been considered a potential target for allergy prevention. Maternal diet may affect, through direct or indirect mechanisms, infant GM, which is associated with a range of allergy outcomes [13,14,15]. High adherence to the Mediterranean diet has been reported to increase GM diversity [16]. Thus, a maternal diet rich in Mediterranean diet-based foods, including vegetables and yogurt, was associated with protective effects for offspring allergies [17]. On the contrary, dietary intake of Western heat-processed foods high in advanced glycation end products (e.g., fried foods, red and processed meat and fruit juice), has been associated with a reduced diversity of GM and the occurrence of pediatric allergies [2]. In addition, the maternal avoidance of allergenic solids foods during pregnancy and lactation have been considered ineffective for CMA prevention and no recommendation by international guidelines have been provided [8]. Indeed, the maternal intake of allergenic solids foods during pregnancy could ensure the placental transmission of inhibitory IgG- allergen immune complexes, reducing the risk of pediatric allergy occurrence [18,19]. Furthermore, no international recommendations for or against the use of prebiotics, probiotics or synbiotics during breastfeeding and lactation alone or in combination with other approaches to prevent pediatric allergies have been formulated [8].

**Figure 1:**
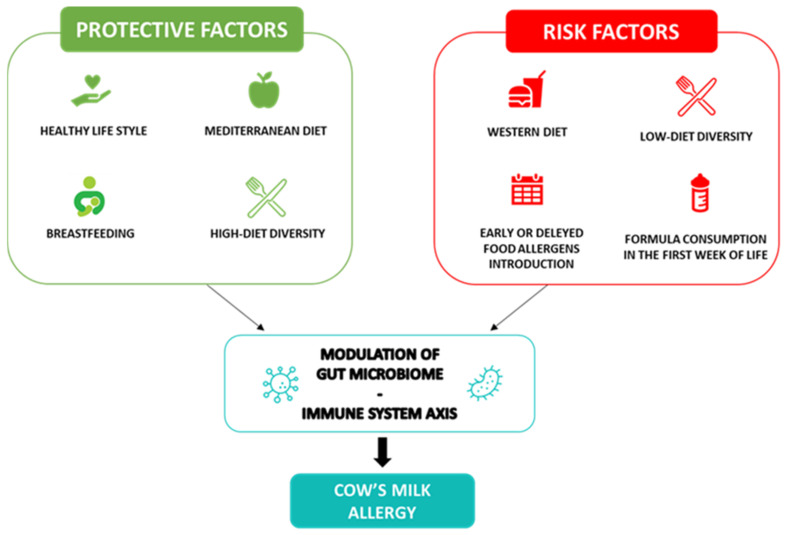
Cow’s milk allergy protective and risk nutritional factors. Legend: The figure depicts the nutritional protective and risk factors in CMA.

Through a positive modulation of the GM and the proper development of the immune system during infancy, breastfeeding is the first nutritional postnatal factor able to protect against allergy occurrence [20]. Several protective mechanisms of breastfeeding have been proposed. Breast milk has anti-allergic immune properties and contains a large amount of biologically active compounds, including lysozyme, lactoferrin, immunoglobulins (Ig)A, IgM, cytokines, nucleotides, microRNAs and hormones that provide passive immunity and could induce oral tolerance to food antigens [21,22,23]. Among the most abundant protective component of breast milk, are the human milk oligosaccharides (HMOs) and prebiotics, resulting in the production of sub-products such as lactate and short-chain fatty acids (SCFAs) and metabolites, able to modulate the immune system function [24]. In particular, the SCFA butyrate enhances the suppressive capacity of regulatory T cells (Treg), suppressing the allergic response and sustaining immune tolerance to allergens in the offspring [25,26]. Butyrate in human milk modulates the mechanisms of immune tolerance, including the increase in biomarkers of gut barrier integrity and tolerogenic cytokines in concentrations able to protect against allergy occurrence [27]. Nevertheless, due to the low certainty of evidence, no recommendations for or against using breastfeeding to prevent food allergy or CMA have been provided at the European level [8]. Thus, considering the multiple benefits for infants and mothers, breastfeeding should be encouraged wherever possible, as stated by most scientific society guidelines [28]. Notably, guidelines from the European Academy of Allergy and Clinical Immunology (EAACI) recommend avoiding supplementation with cow’s milk formula in the first week of life. Other possible temporary supplementary options for breastfed infants could include donor breast milk, amino acid or hydrolyzed formula [8]. Regarding the introduction of complementary foods in infants’ diets for allergy prevention, the European guidelines recommend the importance of not avoiding the intake of potentially allergenic foods during weaning, emphasizing that there is no reason for delaying their introduction after 12 months nor for an early introduction <4 months [29]. Evidence supports the role of early exposure to potential allergens in the development of immune tolerance [30,31]; indeed, the regular ingestion of food allergens between 4 and 6 months of life can lead to immune tolerance and alter the immunological responses to food antigens; conversely, the skin passage of food antigens in the condition of inflammation, before the achievement of immune tolerance, can lead to sensitization to food allergens [32]. In addition, the infants’ diet, influencing the GM composition and function, could have a pivotal role in protecting against the occurrence of food allergy. Evidence has shown that a high diet diversity and the introduction in the first year of life of fruits, vegetables, yogurt and fish, through a fecal increase in the tolerogenic metabolite butyrate, is associated with protection against the development of allergies, even in later stages of life [33,34]. Figure 1 shows a summary of the protective and risk nutritional factors in CMA occurrence.

## 3. Nutritional Strategies for the Management of Cow’s Milk Allergy

In pediatric patients with a confirmed CMA diagnosis, international guidelines recommend a strict cow’s milk protein elimination diet as the only therapeutic strategy to treat CMA. Obviously, the first aim of the elimination diet is to prevent an allergic reaction in CMA patients; but for specialists working in the FA field, avoiding nutritional deficit and ensuring optimal body growth are pivotal aspects that need to be considered [35,36,37,38]. In Table 1 are reported the nutritional intervention cornerstones in CMA pediatric patients. Nutritional counseling for CMA management should be based on the parental/patients’ education about accidental exposure prevention, cow’s milk restrictions, use of an alternative formula in non-breastfed CMA patients, supplementation of avoided nutrients and adequate follow-up.

### 3.1. Accidental Exposures Prevention

Another cornerstone of nutritional counseling is educating patients and their parents/tutors to prevent accidental ingestion of the allergen. Since cow’s milk is used in many products such as cakes, pastry cream, chocolate, candies, etc., and it represents the main food of child nutrition, especially in the first years of life, its elimination from the diet could be even more difficult [39]. For this reason, accidental exposures could be common and should be prevented. Parents/patients need to be educated regarding:

(1) Contamination: They should be careful about the contact or contamination of foods with cow’s milk protein, especially in places such as bakeries and restaurants, laying out side-by-side of these foods or using the same knife while cutting or using the same fork while serving increases the risk of contamination;
(2) Food labels: All ingredient labels on food packages should be read carefully. Foods containing casein, whey, lactalbumin, albumin, etc. should be avoided [40]. There is no consensus yet on the restriction of foods containing advisory labeling such as “may contain milk” because the allergic risks of these products are not yet fully known and the amount of cow’s milk protein contamination of them are variable [41];
(3) Cross reactions: Due to the high cross-reactivity with cow’s milk protein of sheep, goats, buffalo, ibex, deer, donkey, camel and horse milk, parents should be aware of cross-reactions that may occur and should strictly avoid the consumption of these alternative milks;
(4) Non-food products: Drugs, cosmetics and supplements may contain cow’s milk. The labels of these products should also be read carefully.

### 3.2. Cow’s Milk Protein Elimination Diet

The CMA treatment is based on the exclusion from the diet of cow’s milk and milk derivatives (dairy products). In breast-fed CMA patients, lactating mothers should be encouraged to continue breastfeeding following a cow’s milk protein-free diet. Appropriate nutritional counseling to completely exclude milk and hidden sources of cow’s milk protein from lactating women’s diets and to ensure their nutritional needs (including calcium and vitamin D supplementation) should be performed [42]. In cases where breast milk is not possible and till the second year of age, formula based on cow’s milk and milk from other mammals (e.g., goat’s milk, sheep’s milk, etc.) should be strictly avoided and a hypoallergenic formula should be chosen [42]. In non-breastfed infants, an extensively hydrolyzed formula (eHF) of casein or whey proteins should be the first choice [43]. The use of an amino acid-based formula (AAF) is recommended in patients with severe CMA symptoms or in patients that have no improvement within 2 weeks of using eHF [42]. Formula based on soy protein could be chosen after the establishment of tolerance to soy protein for infants >6 months who do not tolerate the eHF taste or for its more affordability; formula based on rice protein that is partially or extensively hydrolyzed may be considered in infants who refuse or do not tolerate eHF or in vegan families [43].

Since cow’s milk and its derivatives are not only a major source of proteins and fats but also of micronutrients, such as calcium, zinc, riboflavin, magnesium, phosphorus, pantothenic acid, vitamin B12 and vitamin D, a cow’s milk elimination diet without proper substitution of its components could represent a risk for nutritional status of CMA patients [44]. These macros/micronutrients are crucial for infant development and an inadequate elimination diet could lead to serious consequences, including poor growth and malnutrition [45]. Thus, to avoid the high risk of nutritional deficits for CMA patients following an elimination diet, any nutrient supplementation should be considered during nutritional counseling. In particular, calcium supplementation should be performed for mothers of breast-fed CMA patients (e.g., 1000 mg/day), and should be evaluated for children after the introduction of solid foods when milk consumption progressively decreases. Calcium supplementation should be considered for the entire duration of the exclusion diet of non-breastfed infants considering the amount and the composition of the hypoallergenic formula consumed [42]. The vitamin D supplementation should be performed in all patients during the first year of life and should be considered if continuing the administration throughout the entire period of the exclusion diet [46]. In addition to calcium and vitamin D, cow’s milk is an ideal vehicle for the introduction of ω-3-polyunsaturated fatty acids (PUFAs), exerting several beneficial immune-modulatory effects [47]. Low ω-3 PUFA levels in plasma have been reported in CMA pediatric patients [48,49,50]. Their supplementation should be evaluated in CMA patients, also considering the ω-3 PUFAs plasma profile.

The monitoring of a CMA patient’s nutrient intake is of pivotal importance during nutritional counseling to prevent or correct nutritional status alterations. Body growth is determined by genetic factors, and everyone generally follows a certain growth curve if conditions are favorable [51]. Among these factors, diet plays a key role: an adequate diet, in terms of quantity and quality, providing the necessary substrates, can exert an appropriate stimulus to growth [52]. Unfortunately, data on CMA patients’ body growth are conflicting [49,53], paving the way for the hypothesis that alterations of weight and/or length indexes may not only depend on an inadequate elimination diet but also from other causes. In several studies, it has been reported that children with CMA are often at risk of metabolic bone disorders, leading to pathologic conditions such as ricket or osteopenia [54], characterized by a reduction in body bone mineral content, bone mineral density for age, delay of bone age and reduced height for age index [55]. Bone is a mineralized connective tissue in which calcium represents the major component [56] and phosphorus (P) is as a percentage the second mineral component of bone tissue [57]. During the growth period, an adequate intake of calcium is required to maintain a positive calcium balance [58], essential for the development of bones [59] and for optimizing bone mass accretion. The appropriate levels of calcium and phosphorus are crucial for the activity of osteoblasts and osteocytes in the process of matrix mineralization [57]. Patients affected by CMA also showed lower vitamin D concentrations [60]. Vitamin D is known for its role in calcium [61] and phosphorus [62] homeostasis and for optimal skeletal health because it acts as a prohormone essential for the normal absorption of calcium and phosphorus from the gut [63]. With an adequate vitamin D state, intestinal calcium absorption increases from 30% to 40% but without it, the body absorbs no more than 10–15% of the dietary calcium [64]. A deficiency of vitamin D and/or calcium has been observed to lead to a defect in the growth plates and osteoid mineralization, resulting in reduced differentiation of chondrocytes and apoptosis in the growth plate and a consequent increase in osteoid tissue, leading to the appearance of clinical and radiological features of rickets [65]. Chondrocytes both contain the vitamin D receptor (at least in hypertrophic but nonproliferating chondrocytes) and can produce 1,25(OH)2D [66]. Vitamin D is directly involved in endochondral ossification [66]. The process of endochondral ossification affects height. Endochondral ossification occurs within the cartilaginous sketch, where chondrocytes multiply and degenerate; these are later replaced by osteoblasts, which produce bone substance, remain enclosed, and turn into osteocytes. The progression of endochondral ossification and linear growth is regulated by the influence of endocrine/paracrine/autocrine signals and zinc, which mediate a cascade of signals through different pathways [67]. Zinc also has a very important role within the growth cartilage since up to 10% of human genes encode proteins with zinc-binding domains [68]. Several randomized studies have examined the effect of zinc supplementation on child growth, and some of these have concluded that zinc supplementation has a positive effect on children’s growth [69,70,71,72,73], while others have not reported effects [74,75]. Differences obtained may concern variability in study settings, period of supplementation (maternal or infancy/childhood), inclusion or exclusion criteria, dose, duration or type of zinc, presence of iron supplementation or types of results evaluated. Furthermore, another issue for nutritional deficiencies in CMA patients may be because the disease is accompanied by an aberrant inflammatory process that can affect serum concentrations of micronutrients [76]. Inflammation inhibits endochondral ossification through the action of mediators, including proinflammatory cytokines [77]. Major proinflammatory cytokines known to inhibit endochondral ossification include TNF-α, IL-1 (particularly IL-1β), and IL-6 [78]. High concentrations of these cytokines suppress growth by decreasing chondrocyte proliferation while increasing apoptosis [79]. TNF-α is produced endogenously throughout the growth cartilage and can inhibit chondrocyte proliferation, especially in combination with IL-1β [80]. IL-1β induces rapid dedifferentiation of cultured chondrocytes [81] and acts synergistically with TNF-α to inhibit longitudinal bone growth of cultured rats [82].

### 3.3. Proposal of the Most Complete Compounds Supplement for Nutrients Deficiencies Prevention and to Positively Drive the Disease Course in CMA Pediatric Patients

Interventions are required to avoid the harmful consequences of nutritional deficiencies, including nutrition education, fortification and supplementation [83]. An elimination diet does not necessarily affect the growth of children if their diet is adequately supplemented. This underscores the importance of an adequate supplementation of deficient nutrients during the dietary management in CMA children, and in general of FA children [84]. Several nutrient supplements available on the market are selective only for some types of nutrients and are deficient in others. For this reason, based on emerging data in the CMA field, with this review, we would like to propose the most complete nutrient supplements for children affected by CMA for preventing and treating nutritional status alterations and nutritional deficiencies. We propose a complete list of trace elements, vitamins, minerals and essential fatty acids, which are necessary for the well-being of the child, and which are mostly deficient in children affected by CMA. In addition to the nutrients mentioned above, we would like to add to the list specific probiotics, prebiotics and postbiotics which have an important role in children’s health and CMA disease course [85]. Emerging data suggest that GM manipulation with the use of pro-, pre- and postbiotics may have a role in the prevention or treatment of FAs, such as CMA [86,87]. Several studies have examined the effects of probiotic supplementation in CMA treatment. Our group has previously shown that the supplementation with the probiotic *Lactobacillus rhamnosus* GG (LGG) of an extensively hydrolyzed casein formula (EHCF) induced higher tolerance rates to milk in CMA pediatric patients compared with EHCF alone and other formulas [88]. Indeed, the LGG is a butyrate producer, which might modulate the expression of genes involved in the allergic pathway, improving the tolerance to cow’s milk proteins [89]. An additional approach to positively modulate the GM is based on the use of prebiotics [85], such as HMOs, fructooligosaccharides (FOSs), galactooligosaccharides (GOSs) and inulin. HMOs provide health benefits in terms of microbiota composition, modulation of gut epithelial cell responses, and provide immunomodulatory and anti-inflammatory effects [90]. Moreover, HMOs can indirectly increase the production of SCFAs [91], which may improve the main immune defensive functions of the intestinal epithelium [92]. FOSs, GOSs and inulin, through a positive modulation of the GM composition and activity, could elicit beneficial effects on immune system function [93,94]. Furthermore, for the tolerogenic properties of the SCFA butyrate, this postbiotic could be useful for its beneficial effects on the GM-immune system axis [25,26,27].

Other compounds with potential beneficial effects in CMA patients could be quercetin, a flavonoid with several pharmacological properties, including anti-inflammatory and immunomodulatory properties [95], curcumin, which could alleviate allergic symptoms [96], and finally berberine and limonin, bioactive compounds which may suppress the IgE production by human B cells and peripheral blood mononuclear cells, as shown in allergic patients [97].

Starting from this evidence, in Table 2 are listed the compounds that could be necessary for the prevention of nutritional status alterations/deficiencies in CMA pediatric patients and improve the disease course of milk allergies. Obviously, the compounds’ dosage in the supplement will respect the recommended levels of national guidelines and dietary reference values (DRVs) for age [98]. For the compounds, such as probiotics, prebiotics and others, further evidence is needed to establish the recommended amounts to be included in our supplement.

### 3.4. Follow-Up of CMA Patients

The anthropometric parameters assessment represents the most important step of nutritional follow-up since body growth is a sensitive indicator of an adequate nutritional status and optimal intake of energy and nutrients. Weight, length/height and head circumference should be measured, and the values should be reported on the reference growth curves [99]. The nutritional follow-up through the evaluation of body growth parameters and relative percentiles/z-scores should be performed at 1, 2 and 4 months in the first semester of life and every 3 months in the second semester. After the first year, the anthropometric measurements should be performed every 6–12 months. The nutritional intake assessment should be performed at least once a year in CMA patients with normal growth or two or more times per year when patients present an alteration in the body growth pattern [46]. Nutritional status alterations of infants and children are classified by the World Health Organization (WHO) as shown in Table 3. In these cases, a personalized nutritional intervention properly planned and monitored has proven to be an effective strategy to correct body growth alterations [46].

Regarding CMA recovery, several studies indicate that ~80% of CMA patients achieve oral tolerance to milk within 3 to 4 years but this generally depends on different factors that could predict the CMA duration (e.g., the occurrence of other atopic manifestations, such as asthma, rhinitis and eczema and other skin and/or gastrointestinal symptoms) [43] and the CMA-symptoms severity at the diagnosis is associated with worse prognosis for the duration of allergy [100]. Thus, a periodical re-evaluation of tolerance to cow’s milk through oral food challenges according to the guidelines and based on the immunologic mechanism (IgE/non-IgE) is mandatory to prevent CMA patients from continuing unnecessary elimination diets [43]. Surprisingly, more than half of children with CMA may tolerate processed-extensively heated/baked forms of cow’s milk [101]. An oral food challenge with baked milk is the only way to evaluate this tolerance, and it has been shown that patients who tolerate baked milk have a better prognosis of CMA and are more likely prone to tolerate lightly processed forms of cow’s milk in the future [102]. After introducing food products with well-cooked cow’s milk associated with a matrix (e.g., a muffin), it could be useful to stimulate the immune tolerance to milk through the consumption of less heated cow’s milk food products till the uncooked milk, following the “milk ladder” approach [103]. Another approach to induce oral tolerance to milk could be achievable using oral immunotherapy (OIT). This strategy provides the administration of increasing quantities of milk until achieving tolerance. Unfortunately, the OIT procedures should be better standardized, and its efficacy requires further studies [104].

## 4. Conclusions

The prevalence and severity of pediatric CMA have increased dramatically in the last decades under the pressure of environmental factors in genetically predisposed individuals. Among environmental factors, nutrition plays a pivotal role. Diet is one of the most relevant modifiable factors, representing a potential target for the prevention and treatment of CMA. Indeed, the immune system development and function could be modulated by immunonutrition strategies. This narrative review provided an overview of the most scientific-based nutritional strategies for the prevention and management of pediatric CMA.

## Figures and Tables

**Table 1:** Nutritional intervention cornerstones in CMA pediatric patients.

Preventing allergic reaction	Parental/patients’ education about accidental exposures prevention
Avoiding nutritional deficit	To ensure optimal nutrient intake (e.g., Vitamin D, calcium, PUFA) and following a Mediterranean diet rich in prebiotic fiber, fermented foods rich in probiotics, etc.
Ensuring optimal body growth	Adequate follow-up

**Table 2:** Proposal of the most complete compounds supplement for nutrients deficiencies prevention and to positively drive the disease course in CMA pediatric patients.

Minerals	DRVs (7–11 Months)	DRVs (1–3 Years)	DRVs (4–6 Years)	DRVs (7–10 Years)	DRVs (11–14 Years)	DRVs (15–17 Years)
Sodium	NA	NA	NA	NA	NA	NA
Potassium	750 mg/day	800 mg/day	1100 mg/day	1800 mg/day	2700 mg/day	3500 mg/day
Chlorine	NA	1.7 g/day	2 g/day	2.6 g/day	3.1 g/day	3.1 g/day
Magnesium	80 mg/day	170 mg/day	230 mg/day	230 mg/day	250–300 mg/day	250–300 mg/day
**Trace Elements**						
Iron	11 mg/day	7 mg/day	7 mg/day	11 mg/day	11 mg/day	13 mg/day
Zinc	2.9 mg/day	4.3 mg/day	5.5 mg/day	7.4 μg/day	10.7 μg/day	11.9–14.2 μg/day
Copper	0.4 mg/day	0.7 mg/day	1 mg/day	1 mg/day	1.1–1.3 mg/day	1.1–1.3 mg/day
Manganese	0.02–0.5 mg/day	0.5 mg/day	1 mg/day	1.5 mg/day	2 mg/day	3 mg/day
Molybdenum	10 μg/day	15 μg/day	20 μg/day	30 μg/day	45 μg/day	65 μg/day
Selenium	15 μg/day	15 μg/day	20 μg/day	35 μg/day	55 μg/day	70 μg/day
Chromium	NA	NA	NA	NA	NA	NA
Iodine	70 μg/day	90 μg/day	90 μg/day	90 μg/day	120 μg/day	130 μg/day
**Vitamins**						
Vit. A	250 μg RE/day	250 μg RE/day	300 μg RE/day	400 μg RE/day	600 μg RE/day	650–750 μg RE/day
Vit. D3	10 μg/day	15 μg/day	15 μg/day	15 μg/day	15 μg/day	15 μg/day
Vit. E	5 mg/day	6 mg/day	9 mg/day	9 mg/day	11–13 mg/day	11–13 mg/day
Vit. K	10 μg/day	12 μg/day	20 μg/day	30 μg/day	45 μg/day	65 μg/day
Thiamine (Vit. B1)	0.1 mg/MJ	0.1 mg/MJ	0.1 mg/MJ	0.1 mg/MJ	0.1 mg/MJ	0.1 mg/MJ
Riboflavin (Vit. B2)	0.4 mg/day	0.6 mg/day	0.7 mg/day	1 mg/day	1.4 mg/day	1.4 mg/day
Niacin (Vit. B3)	1.6 mg NE/MJ	1.6 mg NE/MJ	1.6 mg NE/MJ	1.6 mg NE/MJ	1.6 mg NE/MJ	1.6 mg NE/MJ
Pantothenic Acid	3 mg/day	4 mg/day	4 mg/day	4 mg/day	5 mg/day	5 mg/day
Vit. B6	0.3 mg/day	0.6 mg/day	0.6 mg/day	1 mg/day	1.4 mg/day	1.6–1.7 mg/day
Folic Acid	80 μg DFE/day	120 μg DFE/day	140 μg DFE/day	200 μg DFE/day	270 μg DFE/day	330 μg DFE/day
Vit. B12	1.5 μg/day	1.5 μg/day	1.5 μg/day	2.5 μg/day	3.5 μg/day	4 μg/day
Biotin	6 μg/day	20 μg/day	25 μg/day	25 μg/day	35 μg/day	35 μg/day
Vit. C	20 mg/day	20 mg/day	30 mg/day	45 mg/day	70 mg/day	90–100 mg/day
**Fatty Acids**						
Alpha-Linolenic acid (ALA)	0.5 E%	0.5 E%	0.5 E%	0.5 E%	0.5 E%	0.5 E%
Linoleic acid (LA)	4 E%	4 E%	4 E%	4 E%	4 E%	4 E%
Arachidonic acid (ARA)	NA	NA	NA	NA	NA	NA
Eicosapentaenoic Acid (EPA)	100 mg/day	100–250 mg/day	250 mg/day	250 mg/day	250 mg/day	250 mg/day
Docosanoic Acid (DHA)	100 mg/day	100–250 mg/day	250 mg/day	250 mg/day	250 mg/day	250 mg/day

Legend: In this table is reported a list of compounds that could be useful for the prevention of nutritional status alterations/deficiencies of CMA pediatric patients, and to improve the disease course of milk allergy. Abbreviations: DRVs: Dietary Reference Values; NA: Not Available; RE: Retinol equivalents; NE: Niacin Equivalents; DFE: Dietary Folate Equivalents; E: Energy.

**Table 3:** Classification of nutritional status alterations of infants and children according to WHO.

Classification	Description
Moderately underweight	Weight-for-age <−2 SD and ≥−3 SD
Severely underweight	Weight-for-age <−3 SD
Moderate acute malnutrition	Weight-for-length/height or BMI-for-age ≤−2 SD and ≥−3 SD, or mid-upper arm circumference ≥115 mm and <125 mm
Severe acute malnutrition	Weight-for-length/height or BMI-for-age <−3 SD, or mid-upper arm circumference <115 mm, or bilateral pitting edema
Moderate chronic malnutrition	Length/height-for-age ≤−2 SD and ≥−3 SD
Severe chronic malnutrition	Length/height-for-age <−3 SD
Moderately wasted	Weight-for-length/height ≤−2 SD and ≥−3 SD
Severely wasted	Weight-for-length/height <−3 SD

Legend: in this table is reported the classification of nutritional status alterations of infants and children according to the WHO. Abbreviations: SD: standard deviations; BMI: body mass index.

## Data Availability

Not applicable.
